# Identification of a Novel Zinc Metalloprotease through a Global Analysis of *Clostridium difficile* Extracellular Proteins

**DOI:** 10.1371/journal.pone.0081306

**Published:** 2013-11-26

**Authors:** Valeria Cafardi, Massimiliano Biagini, Manuele Martinelli, Rosanna Leuzzi, Jeffrey T. Rubino, Francesca Cantini, Nathalie Norais, Maria Scarselli, Davide Serruto, Meera Unnikrishnan

**Affiliations:** 1 Novartis Vaccines and Diagnostics, Siena, Italy; 2 Magnetic Resonance Center, University of Florence, Sesto Fiorentino, Italy; 3 Department of Chemistry, University of Florence, Sesto Fiorentino, Italy; University of Helsinki, Finland

## Abstract

*Clostridium difficile* is a major cause of infectious diarrhea worldwide. Although the cell surface proteins are recognized to be important in clostridial pathogenesis, biological functions of only a few are known. Also, apart from the toxins, proteins exported by *C. difficile* into the extracellular milieu have been poorly studied. In order to identify novel extracellular factors of *C. difficile*, we analyzed bacterial culture supernatants prepared from clinical isolates, 630 and R20291, using liquid chromatography-tandem mass spectrometry. The majority of the proteins identified were non-canonical extracellular proteins. These could be largely classified into proteins associated to the cell wall (including CWPs and extracellular hydrolases), transporters and flagellar proteins. Seven unknown hypothetical proteins were also identified. One of these proteins, CD630_28300, shared sequence similarity with the anthrax lethal factor, a known zinc metallopeptidase. We demonstrated that CD630_28300 (named Zmp1) binds zinc and is able to cleave fibronectin and fibrinogen *in vitro* in a zinc-dependent manner. Using site-directed mutagenesis, we identified residues important in zinc binding and enzymatic activity. Furthermore, we demonstrated that Zmp1 destabilizes the fibronectin network produced by human fibroblasts. Thus, by analyzing the exoproteome of *C. difficile*, we identified a novel extracellular metalloprotease that may be important in key steps of clostridial pathogenesis.

## Introduction

*Clostridium difficile*, a Gram-positive, spore-forming, anaerobic bacterium, is one of the main causes of antibiotic-associated diarrhea worldwide. The clinical outcomes, which are generally referred to as *C. difficile* associated disease (CDAD), range from mild diarrhea to more severe conditions such as pseudomembranous colitis and toxic megacolon [1,2]. In the last decade, new epidemic strains belonging to the BI/NAP1/027 category have emerged, causing an increase in rates and severity of CDAD in North America and Europe [3,4]. Recurrent infections and an increase in antibiotic-resistant strains have made treatment of *C. difficile* infections extremely difficult [5].

The two glucosyltransferase toxins, toxin A and toxin B, are crucial virulence factors of *C. difficile* [6,7]. Following internalization by the gut epithelial cells, these toxins are able to inactivate Rho family GTPases, leading to disruption of the actin cytoskeleton and death of colonocytes, with dramatic consequences on the function of the intestinal epithelial barrier and establishment of a severe inflammatory response [8]. In addition to toxin A and toxin B, a minority of strains produce a binary toxin, also called *C. difficile* transferase (CDT), that ADP-ribosylates actin, causing disruption of the host cell cytoskeleton [9].

Apart from toxins, other factors are important for the establishment of the bacterium in the gut and the development of pathogenesis. It has been shown that some surface proteins of *C. difficile* influence interactions with the host and the outcome of infection. The fibronectin-binding protein Fbp68 is important in adhesion and colonization of *C. difficile* [10]. Flagellar proteins have been reported to be involved in adherence, although their functions during infection are not clear [11,12] The high molecular weight (MW) surface layer protein is involved in adherence of *C. difficile* to host cells [13], while cell wall proteins (CWPs) Cwp66 and Cwp84 have been shown to be important in adherence and degradation of extracellular matrix respectively [14,15]. Also, it is likely that during colonization of the gut *C. difficile* releases not only the known toxins but also other proteins. However, there is little information about proteins secreted by *C. difficile* into its environment. A recent report described Srl, a protein found in culture supernatants that modulates the cell sensitivity to toxins A and B [16].

A systematic identification and functional characterization of *C. difficile* secreted proteins that are exposed on the surface or released in the environment, is central to understand the mechanisms involved in *C. difficile* interactions with the host. Proteomics have been previously employed effectively to identify new pathogenic determinants in various pathogens and to understand differences between various clinical strains [17,18]. Although previous studies have tried to characterize extracellular protein profiles of *C. difficile* [19-21], the precise cellular localization of the proteins identified was unclear due to the presence of cytoplasmic proteins. The goal of this work was to employ proteomics to identify novel extracellular factors that may be relevant in *C. difficile* pathogenesis. Here we describe the identification of *C. difficile* extracellular proteins in bacterial culture supernatants from two clinically relevant isolates using liquid chromatography-tandem mass spectrometry (LC-MS/MS). Moreover, from the analysis of *C. difficile* supernatants, we identified and characterized a novel zinc-dependent metalloprotease, Zmp1, which is able to degrade fibrinogen and fibronectin *in vitro*. 

## Materials and Methods

### Bacterial strains and culture conditions

*C. difficile* clinical isolates 630 and R20291 were used in this study. Bacteria were grown in liquid or solid media at 37°C under anaerobic conditions (80% N_2_, 10% CO_2_, 10% H_2_) in a Don Whitley workstation (Yorkshire, UK). 

For proteomic analysis, bacteria were cultured in chemically defined minimal medium (CDMM), prepared as described previously [22]. Bacteria from glycerol stocks were grown O/N on solid BHI (brain heart infusion) medium supplemented with 5 g/l yeast extract and 1 g/l cysteine. One single colony was inoculated in 5 ml of CDMM and grown O/N to stationary phase. The resulting culture was diluted in 5 ml of the same medium to a starting OD_600_ of 0.05 and grown to mid-exponential phase (0.4-0.8). The culture was diluted again to a starting OD_600_ of 0.05 in 50 ml of the same medium, grown up to mid-exponential phase (0.4-0.8) and used for subsequent sample preparation for mass spectrometry (MS).

### Sample preparation for MS/MS analysis

To analyze the protein content of culture supernatants, after reaching the desired phase of growth, cultures were centrifuged at 3,200 *g* for 10 min. The supernatant was filtered through a 0.22 µm filter to remove any remaining bacteria, and EDTA-free Complete protease inhibitor cocktail (Roche, NJ, USA) and 5 mM EDTA were added. Proteins were precipitated by 10% TCA (trichloroacetic acid) (v/v), 0.04% sodium deoxycholate (w/v) incubating for 3 h on ice, followed by centrifugation at 37,000 *g* for 20 min. The pellet was resuspended in 10% TCA and centrifuged as before. Pellets were washed three times with decreasing amounts of cold absolute ethanol (half volume, 1 ml, 200 µl), then dried in a vacuum concentrator and suspended in 50 mM ammonium bicarbonate. 

Proteins recovered from the pellet were denatured by 0.1% Rapigest® (w/v) (WatersTM, MA, USA) and 5 mM DTT and boiled for 10 min. After cooling, the pH was adjusted to 7.8- 8.5 using ammonium bicarbonate. 1µg of trypsin was added and the digestion reaction was incubated O/N at 37°C. Digested samples were cleaned using OASIS Cartridges HLB (WatersTM) for LC-MS/MS analysis.

### Protein identification by nano-LC-MS/MS

Peptides were separated by nano-LC on a NanoAcquity UPLC system (WatersTM) connected to a Q-ToF Premier Electro Spray Ionization (ESI) mass spectrometer equipped with a nanospray source (WatersTM).  Samples were run as previously described [23]. Protein identification was achieved using MASCOT (version 2.2.1) by searching in a locally curated database combining protein sequence data derived from the *Clostridium difficile* section of the NCBInr database, the total number of sequences and residues being 57,275 and 17,440,799, respectively. The score thresholds for acceptance of peptide identification were ≥48 for trypsin digestion, as defined by the MASCOT scoring. 

### Preparation of *C. difficile* and culture supernatant fractions and immunoblotting

To prepare a total cell extract, strain 630 was grown in Trypticase Yeast extract Mannitol (TYM) to OD_600_ 1.3 (exponential phase in this medium). Cells were harvested by centrifugation and the pellet was washed, resuspended in PBS (phosphate buffered saline) and lysed by freeze-thawing. To isolate the cell wall fraction, containing the S-layer proteins and other proteins present within the cell wall, bacteria were grown in 20 ml of TYM to OD_600_ 1.3. Cells were collected by centrifugation at 3,200 *g*, washed once in PBS and once in Tris-sucrose buffer (10 mM Tris-HCl pH 6.9, 10 mM MgCl_2_, 0.5 M sucrose). The pellet was then incubated in 2 ml of digestion buffer (Tris-sucrose buffer with 250 µg/ml mutanolysin and protease inhibitors) for 2 h at 37°C with gentle agitation. The reaction was centrifuged to separate the supernatant that contains the cell wall fraction from the pellet that contains the protoplast fraction. Protoplasts were resuspended in PBS and lysed by freeze-thawing.

The S-layer fraction, containing only proteins associated to the S-layer, was prepared as described previously [24] after growing bacteria in 50 ml of TYM broth to OD_600_ 1.3. To prepare supernatant fractions, bacteria of strain 630 were grown in TYM to the exponential growth phase (OD_600_ 1.3) and centrifuged at 3,200 *g* to separate the culture supernatant (total supernatant). The total supernatant was ultracentrifuged at 150,000 *g* for 16 h to obtain two fractions, ultracentrifugation pellet (UP) and ultracentrifugation supernatant (US). To prepare total supernatant and US fractions for immunoblot analysis, proteins were precipitated by addition of TCA, as described for LC-MS/MS analysis, and then resuspended in PBS. The UP was immediately resuspended in PBS. 

10 µl of different cellular preparations, concentrated with respect to the culture volume, were analyzed by SDS-PAGE: 10X total extract, 20X protoplast, 10X mutanolysin extract, 100X S-layer extract and 10X supernatant fractions (total supernatant, supernatant and pellet after ultracentrifugation) were probed for CD630_01830, CD630_02370 (FliD) and CD630_23650. 3 times more of each cell fraction and 25 times more of each supernatant fraction were analyzed for CD630_28300 (Zmp1). Samples were separated by SDS-PAGE on a NuPAGE gel (Invitrogen) and transferred onto nitrocellulose membranes for western blot analysis. The membranes were blocked for 1 h with 10% milk in PBS, 0.05% Tween-20 at RT. Primary antibodies used were polyclonal murine antisera raised against recombinant His-tagged CD630_01830, CD630_02370 (FliD), CD630_23650 and CD630_28300 (Zmp1). All the primary antibodies used were diluted 1:1000 and incubated with the membrane for 1.5 h at 37°C. The secondary antibody, goat anti-mouse serum conjugated to horseradish peroxidase (1:5000; Dako), was incubated at RT for 45 min. Super Signal Chemiluminescent Substrate (Pierce) was used for detection of bound antibodies following the manufacturer's instructions.

### Cloning and site-directed mutagenesis of Zmp1

A modified version of pET15 (Novagen), pET15/TEV, was constructed to express N-terminal His-tagged proteins. The multiple cloning site of pET15 was replaced with a His-TEV-ccdB-chloramphenicol cassette amplified from the SpeedET vector using polymerase incomplete primer extension (PIPE). 

The gene fragment encoding Zmp1, corresponding to residues 27–220, was amplified by PCR from *C. difficile* 630 genomic DNA using primers Zmp1-F (5’-CTGTACTTCCAGGGCGATAGTACTA CTATACAACAAAATAAAG) and Zmp1-R (5’-AATTAAGTCGCGTTATTTAGCTA AATTTTGCAAAAAGC). The PCR fragment was cloned into the pET15-TEV vector using the PIPE method (25) to obtain a protein lacking the signal peptide and carrying a 6X His-tag at the N-terminus. Site-directed mutagenesis using the PIPE system was performed to generate the E143A mutant. Primers E143A-F (5’- GAATTACATGCAACAGCACATGCAATAGACC) and E143A-R (5’-GTGCTGTTGCATGTAATTCTAAATTTATTGC) were used to substitute the GAA codon (glutamate) with the GCA codon (alanine). Site-directed mutagenesis with the GeneArt® Site-Directed Mutagenesis system (Invitrogen-Life Technologies) was performed to generate the H146A mutant. Primers H146A-F (5’-GAAACAGCAGCTGCAATAGACCATATAGTATTAAATGAT) and H146A-R (5’-TATTGCAGCTGCTGTTTCATGTAATTCTAAATTTATTGC) were used to substitute the CAT codon (histidine) with the GCT codon (alanine).

### Expression and purification of wild type and mutant recombinant Zmp1

Protein expression was performed in T7 Express *E. coli* cells (New England Biolabs). To produce the unlabeled His-tagged Zmp1 wild type or mutants, the cells were cultured overnight at 30°C in EnPresso medium (Biosilta-Oulu, Finland) following the manufacturer’s instructions. Protein expression was induced with 1 mM IPTG for 8 h at 30°C. To produce ^15^N labeled samples for NMR (nuclear magnetic resonance) studies, the cells were grown at 37°C in M9 minimal medium containing 1 mg/ml of (^15^NH_4_)_2_SO_4_, until OD_600_^~^ 0.7 and then induced with 1 mM IPTG for 3 h at 37°C. The harvested cells were lysed by sonication in binding buffer (20 mM Tris-HCl, 300 mM NaCl, 10 mM imidazole, pH 8.0) and centrifuged. The supernatant was loaded onto a PD-10 gravity flow empty column (GE Healthcare, NJ, USA) packed with 2 ml of Ni-NTA FF resin (Qiagen GmbH, Hilden, Germany) equilibrated with the binding buffer. The protein was eluted with buffer containing 300 mM imidazole. Buffer was then exchanged in 50 mM Tris-HCl, 0.5 M EDTA, 1 mM DTT, pH 8.0 (TEV buffer) and cleavage of the His-tag with the tobacco etch virus (TEV) protease (Invitrogen) was carried out at RT O/N. After digestion, TEV buffer was replaced with binding buffer and a second subtractive IMAC purification was performed on the Ni-NTA column. The purified tag-less protein, collected in the flow-through, was quantitated using a BCA assay (Pierce, Rockford, IL, USA). The final purity of the proteins was checked by SDS-PAGE. To obtain the recombinant Zmp1 apo samples suitable for the activity assays the protein was incubated in 50 mM sodium acetate, 20 mM EDTA, pH 5.0, at 4°C O/N. The buffer was then exchanged with Tris buffer (50 mM Tris-HCl, 150 mM NaCl, pH 7.6) using a PD-10 desalting column (GE Healthcare, NJ, USA). Different metallated forms of Zmp1 were obtained by addition of ZnCl_2_, CuCl_2_ or NiCl_2_ solutions up to the desired final concentration. 

### Production of recombinant proteins and antibodies

CD630_01830, FliD and CD630_23650 coding sequences were amplified by PCR and cloned into pET15/TEV vector using the PIPE method. Proteins lack the signal peptide and contain a 6XHis-tag at the N-terminus. Protein expression was performed in T7 Express *E. coli* cells (New England Biolabs) as described above for Zmp1. Protein purification was performed by one-step IMAC chromatography following the same protocol already described for Zmp1. The final purity of the proteins was checked by SDS-PAGE. Eight mice were immunized three times with 10 µg of recombinant protein per dose. The resulting sera were pooled and used in immunoblotting analysis.

### Differential scanning fluorimetry (DSF)

The shifts in the melting temperature of Zmp1 wild type, E143A and H146A as a function of Zn^2+^, Cu^2+^ or Ni^2+^ concentration were determined with DSF analysis, as described previously [25]. In each well, apo protein was incubated at a concentration of 20 μM in 20 μl Tris buffer, in the presence of ZnCl_2,_ CuCl_2_ or NiCl_2_ in a final concentration ranging from 0 to 0.5 mM. The plate was subjected to a temperature gradient scan (25–95 °C) in a real time PCR machine (Agilent Technologies, Santa Clara, CA, USA). The fluorescence intensity was measured by use of fluorescence Sypro Orange dye (5x final concentration) at different temperatures with excitation and emission wavelengths of 490 and 575 nm, respectively. 

### NMR analysis

To obtain the recombinant Zmp1 apo samples the protein was incubated in 50 mM sodium acetate, 20 mM EDTA, pH 5.0, at 4°C O/N. The buffer was exchanged in 20 mM HEPES, pH 7.2 using a PD-10 desalting column (GE Healthcare, NJ, USA) and the samples were concentrated up to 100 µM by amiconultra-15 10kDa (GE). NMR experiments were acquired at 298K on an Avance 900 Bruker spectrometer working at 900,13 MHz frequency and equipped with a cryogenically-cooled probe. Zinc binding by apo proteins was assessed by titrating the apo wild type, E143A and H146A Zmp1 proteins with ZnCl_2_ solution (10 mM), directly in the NMR tube under N_2_ atmosphere in the presence of 1 mM DTT. The titration was followed through ^1^H-^15^N HSQC NMR spectra.

### *In vitro* proteolytic activity of Zmp1 on fibronectin and fibrinogen

To test for cleavage of fibronectin, 1µM fibronectin from human plasma (Sigma-Aldrich) was incubated at 37°C up to 24 h in Tris buffer in absence or presence Zmp1 (0.48-7.7 µM) and in absence or presence of 0.5 mM ZnCl_2_, NiCl_2_ or CuCl_2_. To test for cleavage of fibrinogen from human plasma, 1 µM fibrinogen was incubated at 37°C up to 24 h in Tris buffer in absence or presence µM Zmp1 (0.015-1 µM) and in absence or presence of 0.5 mM ZnCl_2_. Reactions were analyzed by separation on NuPAGE gels followed by staining with Problue Safe stain (Giotto Biotech).

### NH_2_-terminal sequence analysis of fibrinogen and fibronectin fragments

Aliquots of fibrinogen and fibronectin samples incubated in presence of Zmp1 24h at 37°C were subjected to SDS-PAGE on 4-12% gels and transferred to polyvinylidene fluoride membranes (iBlot® Transfer Stack- Life technologies). The membranes were stained with 1% Ponceau S/ 1.0% acetic acid and the band corresponding to the degraded fibrinogen beta subunit fragment and 3 bands corresponding to degraded fibronectin fragments were excised and sent to Alphalyse A/S (Odense - Denmark) to be subjected directly to multiple cycles of Edman degradation on ABI Procise 494 sequencer. 

### Proteolytic activity of Zmp1 protein on human fibroblasts

IMR-90 cells (Human fibroblasts, ATCC) were maintained in Eagle’s Minimum Essential Medium (EMEM, ATCC) supplemented with 10% heat-inactivated fetal bovine serum (FBS, Gibco) and antibiotics at 37°C in 5% CO_2_.

To study Zmp1 activity, cells were seeded on 8-well chamber slides coated with collagen I (2.5 x 10^4^ per well) (BD BioCoat) and grown for 2 days. Cells were then incubated with 11.6 µM of recombinant Zmp1 or mutant Zmp1 proteins and 0.1 mM ZnCl_2_ for 16 h at 37°C in EMEM without FBS to avoid the interference of plasma fibronectin. After washings, cells were fixed with 3.7% paraformaldehyde. Samples were washed extensively and incubated with rabbit anti-fibronectin antibodies (Sigma-Aldrich) for 1 h at RT. After multiple washings, samples were incubated with Alexa Fluor 568 goat anti-rabbit IgG. Glass coverslips were mounted with ProLong^®^ Gold antifade reagent with DAPI and analyzed with a Zeiss LSM710 confocal microscope.

For quantitation of fibronectin degradation, as before, IMR-90 cells (seeded equally at 2.5 x 10^4^/well) were cultured on chamber slides for 3 days, followed by incubation with 11.6 µM of recombinant Zmp1 or mutant Zmp1 proteins and 0.1 mM ZnCl_2_ for 16 h at 37°C in EMEM. After repeated pipetting, 100 µl cell culture supernatants were collected from each well and probed with 1:1000 anti fibronectin (Sigma). 

## Results

### Identification of extracellular proteins from *Clostridium difficile* culture supernatants

In order to comprehensively identify proteins present in extracellular milieu during *C. difficile* growth, we analyzed culture supernatants prepared from two clinical *C. difficile* strains, 630 and R20291, grown in chemically defined medium (CDMM), using a shotgun proteomic approach. After bacterial pellet removal, proteins present in the supernatant were precipitated, digested with trypsin and analyzed by LC-MS/MS. The proteins detected in the supernatant were derived only from vegetative cells, as the percentage of spores present in the growth culture was less than 0.01%.

**Figure 1:**
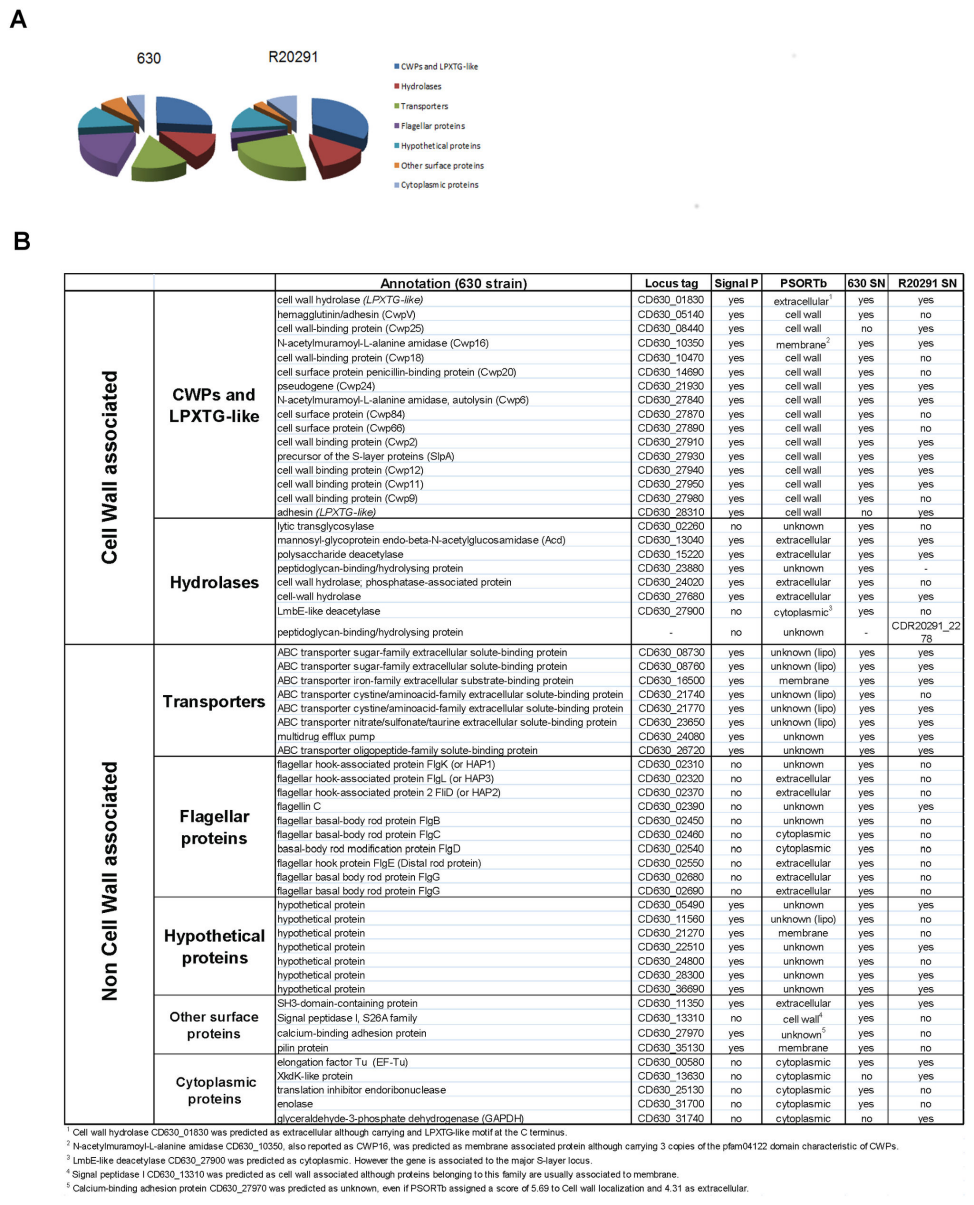
Identification of extracellular proteins from *Clostridium difficile* culture supernatants. (**A**) Family distribution of putative surface/released proteins detected in 630 and R20291 culture supernatants. Proteins identified have been divided in families based primarily on the presence of motifs for association to the peptidoglycan and secondly on the presence of conserved functional domains. (**B**) Proteins detected by LC-MS/MS in *C. difficile* 630 and R20291 culture supernatants. Presence of a signal peptide motif was predicted with SignalP 4.0 using the 630 amino acid sequence (except for CDR20291_2278, for which a sequence with amino acid identity >40% is not present in 630). Cellular localization was predicted by PSORTb 3.0 using the 630 amino acid sequence (except for CDR20291_2278). Detection in 630 or R20291 culture supernatants is indicated by ‘yes/no’.

Analysis of 630 and R20291 culture supernatants led to the identification of a total of 58 unique proteins (6 biological replicates for strain 630, and 3 biological replicates for strain R20291) (Figure 1A and B, Figure S1). Among these, 25 proteins were identified from both strains, while 28 were detected only in 630 and 5 only in R20291. All the genes (except CD630_23880 and CDR20291_2278) encoding the proteins detected in 630 and R20291 are present in both strains, indicating that the lack of detection in one of the two strains may be due to the fact that the protein is not expressed or that it is expressed less under the conditions tested.

The identified proteins were classified into two main classes depending on the predicted association to the cell wall (Figure 1A and B). The group comprising cell wall associated proteins contains 24 proteins. Among these, 16 proteins have motifs responsible for their association to *C. difficile* peptidoglycan, which are either the cell wall binding repeat 2 domain (pfam04122), characteristic of the family of cell wall proteins (CWPs) [24,26] or a LPXTG-like motif that is thought to mediate covalent attachment to the peptidoglycan by sortases, as reported for other Gram-positive bacteria [27,28]. Among the CWPs identified in the supernatant, the only ones which have been previously described are CwpV [29], Cwp84 [15,30], Cwp66 [14], Cwp2 [13,31], SlpA [24,32]. Remarkably, Cwp24 was identified in both 630 and R20291 supernatants, although the corresponding gene has recently been annotated as a pseudogene in the reference 630 genome [33]. The remaining 8 proteins are putative hydrolases likely involved in cell wall metabolism, including the previously characterized autolysin Acd [34].

The group of non-cell wall associated proteins comprises 34 proteins which can be further divided in the following 5 families: transporters (8), flagellar components (10), hypothetical proteins (7), other surface-predicted (4) and cytoplasmic proteins (5) (Figure 1) Most of the identified transporters are solute binding subunits of ABC transporters predicted to be involved in the uptake of sugars, amino acids, peptides or inorganic ions. For five of them, a putative lipobox motif was predicted by DOLOP (http://www.mrc-lmb.cam.ac.uk/genomes/dolop/analysis.shtml). Flagellar components were also a major protein family detected in the culture supernatants. While the major flagellar subunit (FliC) [35] was present in both strains, other proteins predicted to be part of the basal body, hook and cap structures were identified only in 630. It is possible that R20291 flagella are less easily detached from the cell, as compared with 630, or their components are less expressed in the growth conditions tested in this study. Seven proteins annotated as hypothetical were identified. Although cellular localization could not be assigned for most of them by bioinformatics with PSORTb (Figure 1B), a cleavage site for signal peptidase could be predicted by SignalP for all of them (Figure 1B). Our results highlight the importance of experimental analyses to improve the performance of software designed for protein localization prediction.

Only five cytoplasmic proteins were identified. Among these proteins, EF-Tu, GAPDH and enolase have been very often associated to the surface of Gram-positive bacteria [23,36]. With the exception of the EF-Tu (CD630_00580), which was identified in 3 out of 9 experiments, all other cytoplasmic proteins were identified in only one of the 9 experiments. Moreover the number of peptides identified for each cytoplasmic protein was no more than 2 (Figure S1). These data indicate extremely low levels of bacterial cell lysis in the samples analyzed. We did not detect the *C. difficile* toxins A and B in the supernatants, as these toxins are not produced in the conditions we used for our experiments, as described in previous reports [21].

Thus, our data show the extracellular localization of different classes of proteins in culture supernatants from *C. difficile* strains. We also detect new extracellular unknown hypothetical proteins that may have potential roles in *C. difficile* physiology and pathogenesis. 

### Cellular localization of proteins identified by proteomics

In order to clarify the subcellular localization of the proteins found in 630 culture supernatants, antibodies against the recombinant forms of 4 proteins representing different protein classes, a cell wall associated LPXTG-like motif containing protein (CD630_01830, MW-37 kDa), a transporter protein (CD630_23650, MW-39kDa), a flagellar protein, FliD [37] (CD630_02370, MW-58 kDa) and a hypothetical protein (CD630_28300, MW-24 kDa), were generated in mice and used to study the presence of the proteins in the culture supernatants and in other cellular fractions by immunoblotting. These studies were performed in a complex medium, TYM, which may represent a more physiological condition.

**Figure 2:**
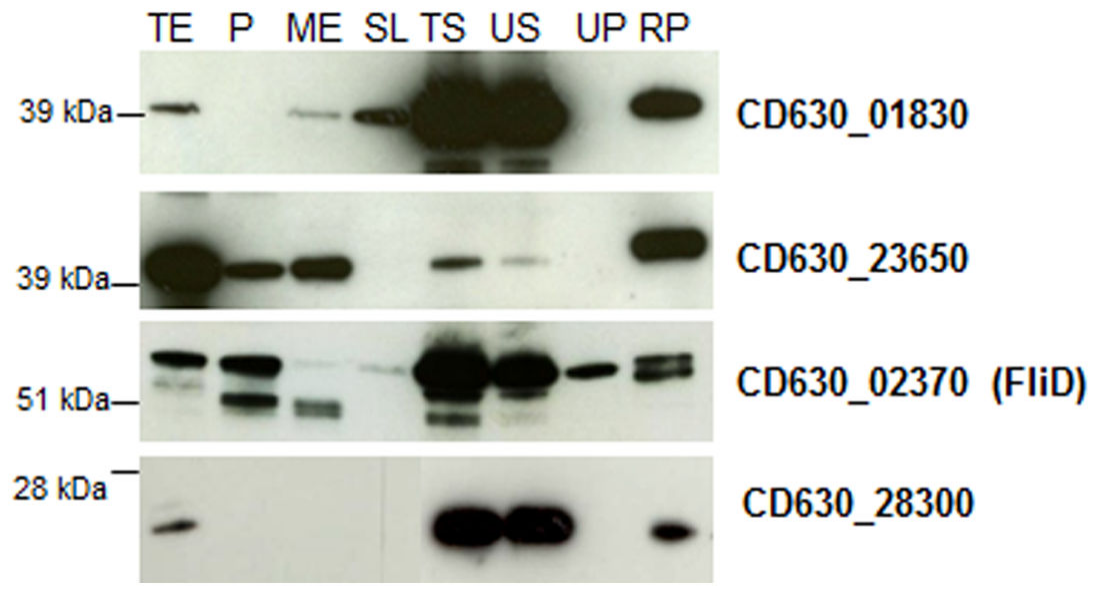
Cellular localization of *C. difficile* 630 proteins detected by LC-MS/MS. Immunoblotting analysis of cell and supernatant fractions prepared from exponential phase cultures with antisera generated against CD630_01830, CD630_23650, CD630_02370 (FliD) and CD630_28300. Cell fractions: total extract (TE), protoplast (P), mutanolysin extract (ME), S-layer extract (SL). Supernatant fractions: total supernatant (TS), supernatant after ultracentrifugation (US), ultracentrifugation pellet (UP). 10 or 50 ng of each recombinant protein (RP) was loaded as control.

In agreement with the proteomic data, the presence of the four proteins in culture supernatants was confirmed. In order to understand if the proteins were released in the supernatant as high molecular weight structures, supernatants were subjected to high-speed centrifugation. Both the ultracentrifugation pellet (UP) and the corresponding ultracentrifugation supernatant (US) were tested by immunoblotting analysis. The proteins CD630_01830, CD630_23650 and CD630_28300 were found in the US and not in the UP (Figure 2), indicating that these proteins were not derived from any membranous or cell wall fragments released from the cells. On the contrary, FliD was also found in the UP, suggesting that entire flagellar structures may be released into the medium. In accordance to the bioinformatic predictions, CD630_01830 was found in the S-layer (SL) and mutanolysin extract (ME) preparations, which are expected to contain proteins associated with the cell wall, while the putative lipoprotein CD630_23650 was found in the protoplast (P) and mutanolysin extract. The flagellar cap protein FliD, on the other hand, was largely found as expected only in the protoplast cell fraction (Figure 2). Besides the supernatant, CD630_28300 was detected in the total extract fraction, but not in the surface fractions, suggesting that it is released from the bacterium rather than surface-associated. 

### CD630_28300 is a putative zinc metallopeptidase

**Figure 3:**
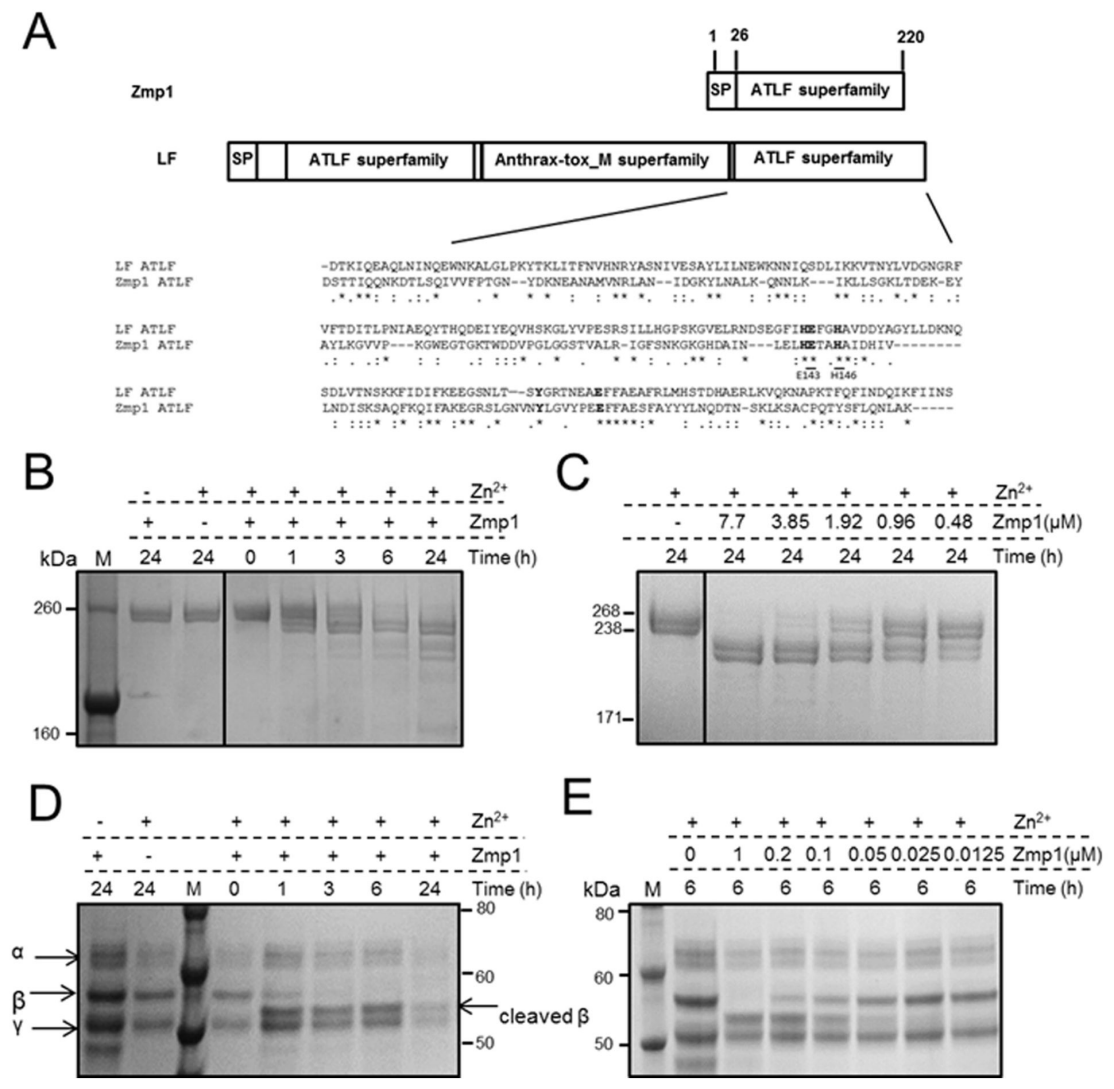
Zmp1 is a zinc-dependent protease with fibrinogen- and fibronectin-cleaving activity. (**A**) Zmp1 shares sequence similarity with the C-terminal ATLF catalytic domain of anthrax lethal factor. Residues previously involved in the zinc proteolytic activity (indicated in bold) are conserved. (**B**) Time-dependent proteolytic activity of recombinant Zmp1 on fibronectin. 1µM fibronectin from human plasma was incubated for 24 h at 37°C with 7.7 µM Zmp1 in the presence of 0.5 mM ZnCl_2_. At 0, 1, 3, 6 and 24 h after incubation, 1 µg of fibronectin was analyzed by SDS-PAGE followed by Coomassie-blue staining. Integrity of fibronectin in the absence of Zmp1 was verified after 24 h of incubation in the same conditions. (**C**) Concentration-dependent activity of Zmp1 on fibronectin was observed after incubation of different concentrations of Zmp1 with 1 µM of fibronectin for 24 h at 37°C in the presence of 0.5 mM ZnCl_2_. 1 µg of fibronectin was analyzed by SDS-PAGE and Coomassie-blue staining. (**D**) Time-dependent proteolytic activity of recombinant Zmp1 on fibrinogen. 1 µM fibrinogen was incubated for 24 h at 37°C with 1 µM Zmp1 in the presence of 0.5 mM ZnCl_2_. At 0, 1, 3, 6 and 24 h after incubation, 5 µg of fibrinogen was analyzed by SDS-PAGE followed by Coomassie-blue staining. Integrity of fibrinogen in the absence of Zmp1 was verified after 24 h of incubation in the same conditions. (**E**) Concentration-dependent activity of Zmp1 on fibrinogen was observed after incubation of different concentrations of Zmp1 with 1 µM of substrate for 6 h at 37°C in the presence of 0.5 mM ZnCl_2_. 5 µg of fibrinogen was analyzed by SDS-PAGE and Coomassie-blue staining.

As our aim was to study previously uncharacterized proteins expressed extracellularly by *C. difficile*, we examined further the unknown hypothetical proteins detected in culture supernatants by BLAST analyses (Table 1). Interestingly, we found that a BlastP search of CD630_28300 against all the proteins in the NCBI database revealed similarity to the lethal factor (LF) of *Bacillus anthracis*. Alignment of CD630_28300 to the C-terminal, catalytically active, anthrax toxin lethal factor (ATLF) domain of anthrax lethal factor (LF) showed that the two sequences share an amino acid identity of 22%, with conservation of residues involved in proteolytic activity in LF and other zinc proteases, including the HEXXH motif (Figure 3A) [38]. CD630_28300 was detected in both 630 and R20291 (named CDR20291_2721) culture media and is conserved among the *C. difficile* genomes reported in the NCBI database with an amino acid identity ranging from 98 to 100%.

**Table 1:** General features of the hypothetical proteins found in both 630 and R20291 supernatants.

**Locus tag**	**Length (aa)**	Conserved domains*	N-terminal signal peptide**
CD630_05490	136	none	yes
CD630_11560	360	Tranglutaminase-like superfamily**^*a*^** (1.13e-07)	lipobox LxxC
CD630_21270	274	none	yes
CD630_22510	118	none	lipobox VxxC
CD630_24800	279	G5 domain**^*b*^** (4.72e-05)	yes
CD630_28300	220	Anthrax lethal factor (4.0 e-48)	yes
CD630_36690	191	GerMN domain**^*c*^** (1.86e-10)	lipobox AxxC

Footnotes: *Conserved domains have been identified running BLAST against the non-redundant GenBank protein database. In parentheses the E values of corresponding alignments are reported.

** The presence of the N-terminal signal peptide has been assessed with PSORTb software (http://www.psort.org/psortb/).

^a^ Transglutaminases are a family of enzymes that catalyze protein crosslink reaction by establishing epsilon-(gamma-glutamyl) lysine isopeptide bonds [62].

*^b^* The G5 domain has been found in a wide range of extracellular proteins, including M26 peptidases and proteins involved in metabolism of bacterial cell walls [63].

*^c^* This domain has been detected and characterized in the putative peptidoglycan binding protein GerM of *Bacillus subtilis* [50].

### CD630_28300 displays a zinc-dependent cleavage of fibronectin and fibrinogen

In order to study the cation-binding ability and proteolytic activity of CD630_28300, a tag-less recombinant protein was produced in *E. coli*. The homogeneity of the purified protein was checked by SDS-PAGE. The protein exhibited a single band on SDS-PAGE with relative mobility consistent with its expected molecular mass (Figure S2).

The protein was incubated with EDTA with low pH to remove any bound divalent cation and differential scanning fluorimetry (DSF) analysis was performed to investigate its ability to bind zinc and other divalent cations. DSF assay showed a shift in melting temperature upon addition of Zn^2+^, Cu^2+^ or Ni^2+^, indicating that the apo-protein is able to bind all these metals (Figure S3), and that the binding to metals stabilizes the protein. Binding to metals was also confirmed by NMR analysis (data not shown).

To understand if the recombinant protein had proteolytic activity, we tested it on casein and gelatin substrates by zymography and FRET (fluorescence resonance energy transfer), and on MAPKKide, a synthetic peptide containing a cleavage site for anthrax lethal factor, by FRET. No activity was detected in any of these assays (data not shown). These data are in accordance with our observation that only few of the residues important for substrate accommodation in the catalytic pocket of LF [39] are conserved in CD630_28300 (Figure 3A), and are suggestive of a proteolytic activity that is distinct with respect to LF.

As other bacterial metalloproteases have been previously reported to be able to degrade extracellular matrix components [40], we examined if CD630_28300 could cleave extracellular matrix (ECM) components *in vitro*. We tested collagen types I, II, III, IV, V and VI, fibrinogen and fibronectin. While none of the collagens were cleaved (data not shown), human plasma fibronectin and fibrinogen were cleaved upon incubation with CD630_28300 *in vitro* at 37°C (Figure 3B-E). The proteolytic effect was time and concentration dependent, with minimal activity observed at 1 h and complete cleavage observed after 6 h for fibrinogen (Figure 3D and 3E) and 24 h for fibronectin (Figure 3B and C). While high Zmp1 concentrations (3.85-7.7µM) were required for activity with fibronectin after 24 h, concentrations of 0. -1µM were sufficient for proteolytic activity with fibrinogen after 6 h. For fibrinogen, the activity was observed as a reduction in molecular weight of the band corresponding to Bβ-chains (Figure 3D, E). Zmp1 activity on both substrates was dependent on the presence of Zn^2+^ (Figure 3B and 3D). Also, upon testing CD630_28300 proteolytic activity in the absence of metals or in the presence of Cu^2+^ or Ni^2+^, we observed the highest activity in the presence of Zn^2+^, in accordance with its zinc-binding function (Figure S4). These data demonstrate that CD630_28300, which we have named zinc metalloprotease 1 (Zmp1), is a novel *C. difficile* zinc-dependent protease. 

The fibrinogen N-terminal sequence established by the automated Edman degradation procedure was Ala-Pro-Pro-Pro-Pro-Ile-Ser-Gly-Gly-Gly, suggesting that the cleavage happens between Pro61 and Ala62 at the N-terminus of the β-chain. The N-terminal sequencing of the tested fibronectin fragments was unsuccessful, perhaps due to the presence of multiple cleaved fragments in the bands sequenced and/or the presence of pyroglutamic acid residues at the N-terminus which block Edman degradation [41].

### Residues E143 and H146 are important for Zmp1 catalytic activity

To further analyze the residues that are involved in Zmp1 catalytic activity, we generated mutants in the HEXXH motif, which is considered a fingerprint for zinc metalloproteases. This motif has been characterized in anthrax lethal factor as well as in other toxins, such as the tetanus and botulinum neurotoxins [42]. In this motif, the imidazole rings of the two histidine residues are part of the first shell of zinc coordination, while the glutamate carboxylate binds the water molecule implicated in the hydrolytic reaction [43]. 

We generated two mutants of Zmp1, E143A and H146A, by site-directed mutagenesis. The purity of mutant recombinant proteins was checked by SDS-PAGE. Both mutants exhibited a single band on SDS-PAGE with the same molecular weight as the wild type protein (Figure S2).

**Figure 4:**
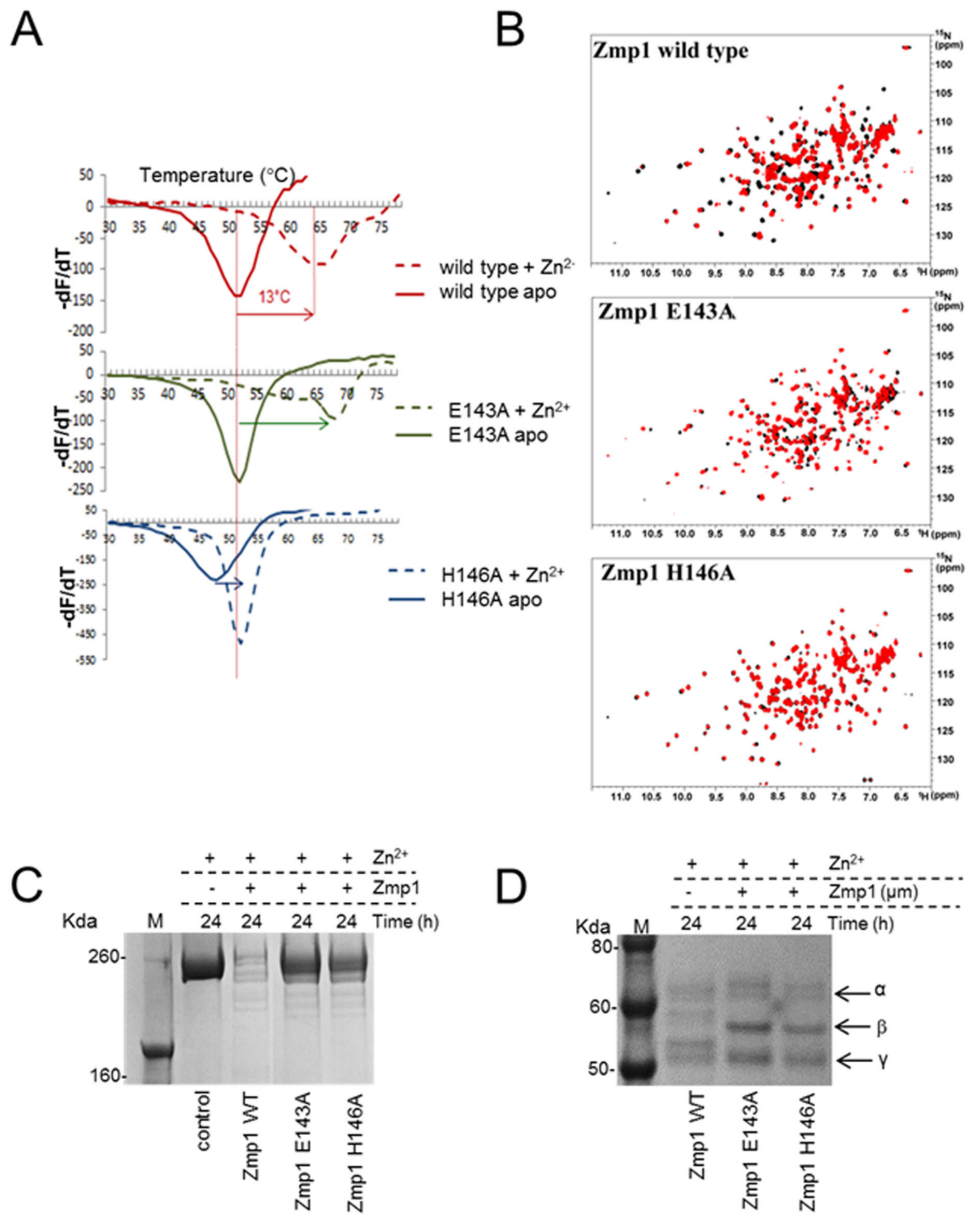
Impaired fibronectin and fibrinogen-cleaving activities of the E143A and H146A Zmp1 mutants. (**A**) Differential scanning fluorimetry of wild type, E143A and H146A recombinant proteins in the absence or presence of 1:16 Zn^2+^. The minimum of the derivative is the melting temperature. Shifts in melting curves for the wild type and mutant proteins are indicated with arrows. (**B**) NMR analysis of wild type, E143A and H146A. ^1^H^15^N HSQC spectrum of apo (black) and in the presence of zinc (red) of Zmp1 wild type, H146A and E143A. (**C**) Decreased ability of E143A and H146A Zmp1 mutants to cleave fibronectin. 1 µM fibronectin from human plasma was incubated for 24 h at 37°C with no Zmp1 (control) or with 7.7 µM of wild type (WT), E143A or H146A Zmp1 in the presence of 0.5 mM ZnCl_2_. 1 µg of fibronectin was analyzed by SDS-PAGE and Coomassie-blue staining. (**D**) Inability of E143A and H146A Zmp1 mutants to cleave fibrinogen. 1 µM fibrinogen was incubated for 24 h at 37°C with 1 µM of wild type (WT), E143A or H146A Zmp1 in the presence of 0.5 mM ZnCl_2_. 1 µg of fibrinogen was analyzed by SDS-PAGE and Coomassie-blue staining.

Stability and zinc-binding ability of these mutants were evaluated by differential scanning fluorimetry (DSF) and nuclear magnetic resonance (NMR) analyses (Figure 4A and 4B). In the DSF assay, both Zmp1 mutants showed a melting temperature comparable to the wild type, indicating that mutations do not affect protein stability. The E143A mutant was able to bind zinc, as demonstrated by an increase in melting temperature observed upon addition of this metal. On the contrary, in the H146A mutant, the ability to bind zinc was completely lost (Figure 4A). NMR analysis showed that wild type and E143A Zmp1 could be metallated by an excess of ZnCl_2_ solution, while H146A was not able to bind zinc, in agreement with DSF data (Figure 4B). Binding of Zn^2+^ was confirmed by atomic absorption spectroscopy. During zinc addition to the wild type and E143A proteins, the ^1^H-^15^N HSQC spectra showed two sets of signals arising from the apo and the zinc-bound forms, indicating that exchange between the two forms is slow on the NMR time scale. The titration was conducted until spectral changes were no longer observed. ^1^H-^15^N HSQC spectrum of zinc-bound wild type and E143A showed well-dispersed resonances indicating that metal binding does not affect the overall fold. These mutants also showed highly reduced activity on fibronectin, as compared to wild type Zmp1 (Figure 4C), indicating that residues E143 and H146 are both important for catalytic activity. To rule out that this residual activity was due to an *E. coli* contaminant protease, an unrelated recombinant protein purified in a similar manner was incubated with fibronectin, but no cleavage of fibronectin was observed (Figure S5). Both mutant proteins showed no activity on fibrinogen in the presence of zinc, confirming that residues E143 and H146 are both important for the catalytic activity (Figure 4D). 

### Zmp1 destabilizes the fibronectin network produced by human fibroblasts

**Figure 5:**
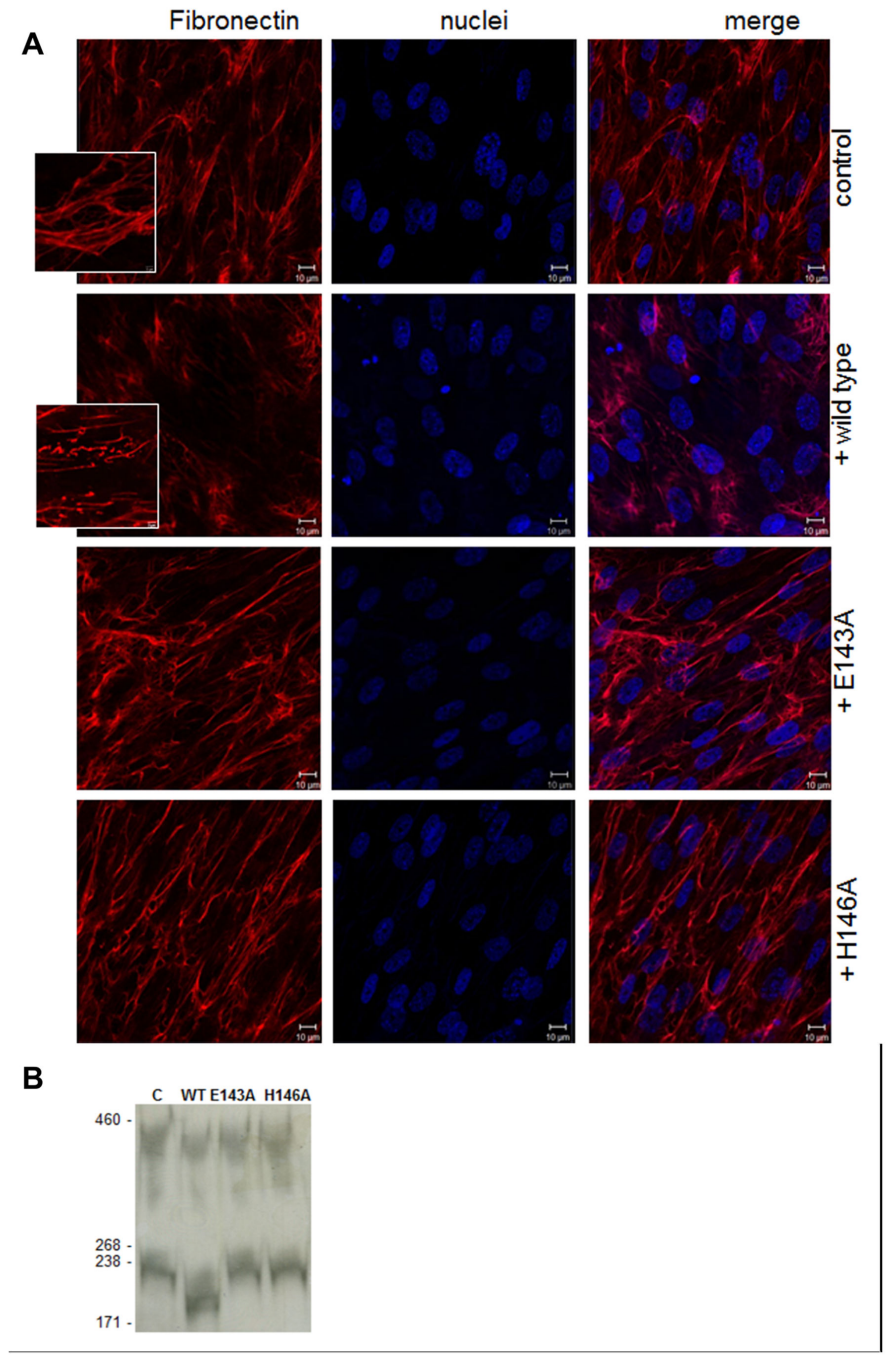
Proteolytic activity of Zmp1 protein on native fibronectin produced by cultured human fibroblasts. (**A**) IMR-90 human fibroblasts were incubated with 11.6 µM of wild type, E143A or H146A Zmp1 protein for 16 h as described in Methods. Fibronectin was labeled using anti-fibronectin followed by Alexa568-conjugated secondary antibodies (red) and nuclei were stained with DAPI (blue). Control cells were incubated with an equivalent volume of buffer for the same time period. Destabilization of fibronectin was observed upon treatment of cells with Zmp1, as also highlighted in the inset. The images shown are representative of 3 independent experiments. (**B**) Immunoblotting analysis of culture supernatants from IMR-90 cells treated with 11.6 µM of wild type, E143A or H146A Zmp1 protein for 16 h. 100 µl undiluted supernatants from each well were probed with anti fibronectin.

We examined if the ability to cleave fibronectin *in vitro* was relevant also in a cell model resembling the ECM components produced by host cells. We used a human fibroblast cell line (IMR-90) producing a complex fiber organization mainly composed of fibronectin and collagens. Upon incubation of the cells with wild type Zmp1 we found that the discrete fibronectin network observed in the untreated cells was destabilized (Figure 5); particularly, we observed the appearance of unstructured fibers of cellular fibronectin indicative of proteolytic activity of the protein (inset in Figure 5A). This effect is zinc-dependent since the fiber disorganization is maximized by the addition of ZnCl_2_ in the culture, whereas a minor effect is observed in the absence of the cation in the medium (data not shown). E143A and H146A mutations in Zmp1 abrogate the observed destabilization of the fibronectin network (Figure 5A). Immunoblotting analysis of cell supernatants incubated with wild type Zmp1, E143A or H146A mutants with anti-fibronectin confirmed that the cells treated with the native Zmp1 show degradation of fibronectin, while the cells treated with medium (control) or with the E143A and H146A mutants show a fibronectin band of the expected size (230-250kDa) (Figure 5B).

## Discussion

Although extracellular proteins are known to mediate events involved in the gut pathogenesis during clostridial infections [13,44], not many cell surface or secreted proteins of the nosocomial pathogen *C. difficile* have been identified or functionally characterized. In a careful analysis of the culture supernatants of clinically relevant strains of *C. difficile*, we found that the exoproteome comprises largely of surface-associated proteins. Further analysis of one of the hypothetical proteins of unknown function that we detected in the extracellular milieu revealed a new zinc-dependent metalloprotease which is able to cleave fibronectin and fibrinogen.

A recent proteomic analysis of culture supernatants from clinical *C. difficile* isolates reported the presence of several putative surface-associated proteins, however, along with the presence of a large number of cytoplasmic proteins [20]. The presence of cytoplasmic proteins, which is a common indicator of lysis, is found in several proteomic studies examining the exoproteomes and complicates the interpretation of the cellular localization of proteins [17,18]. Our work suggests that the proteins we detect in culture supernatants are indeed extracellular, as the preparations we used almost completely lacked proteins of predicted cytoplasmic origin. Among the proteins we detect in supernatants, the Cwp84 cysteine protease and SlpA, the precursor of the two major S-layer proteins, have been well studied and previously reported to be present in culture supernatants [31]. The largest known CWP, CwpV, which promotes *C. difficile* aggregation, was also detected [29]. Several proteins that we find, such as Cwp84, SlpA, FliC, FliD, Cwp2, Cwp18, Cwp66, CD630_26720 and CD630_08730, have been shown to be immunoreactive to human sera [45,46]. In addition to known proteins, our analysis reports many other uncharacterized CWPs, hydrolases, transporters and hypothetical proteins. Further studies of these proteins could provide insight into both bacterial physiology and pathogenesis.

Surface proteins have been reported from culture supernatants of other Gram-positive bacteria [47,48]. The cleavage of surface proteins by proteases has been previously proposed as a mechanism by which proteins are released from the surface in bacteria such as *Streptococcus pyogenes* [49]. The mechanisms controlling the release of surface associated proteins in *C. difficile* are at present unclear. *C. difficile* encodes at least two cysteine proteases, Cwp84 and Cwp13, which are involved in surface maturation [30,31]. A possibility is that these, other reported or yet unidentified proteases are involved in cleavage of some surface proteins of *C. difficile*. Indeed, surface proteins may be released or shed into the extracellular environment, perhaps as a result of abundant expression on the surface. It does not appear to be induced by our culture conditions (growth in minimal medium) *in vitro*, as we see a predominance of surface-associated proteins also in supernatants derived from bacterial growth in various rich culture media tested (data not shown). Presence of surface-associated proteins in the extracellular milieu may suggest that *in vivo*, during infection, such proteins are not just associated to the bacterial surface but may also act distally. 

The global analysis of *C. difficile* supernatants resulted in the identification of seven unknown hypothetical proteins. Based on similarities to known domains or proteins, we find some proteins that may be involved in clostridial pathogenesis. One such protein, CD630_36690, contains a GerMN domain which was previously associated with cell development proteins [50]. The *Bacillus subtilis* GerM protein, which contains a duplicated GerMN domain, was shown to be important for both sporulation and spore germination [51]. The other interesting protein was CD630_28300, which had sequence similarity to the catalytic domain of the *Bacillus anthracis* lethal factor, a well- characterized zinc metalloprotease. We have named CD630_28300 as Zmp1 and have characterized it further in our study.

Zinc metalloproteases are secreted by a variety of bacterial pathogens [40]. Many of these bacterial enzymes have been shown to degrade extracellular matrix molecules such as collagens, fibronectin and mucins. Examples include the metalloproteases produced by *Vibrio* and *Serratia* species [52,53]. Other zinc proteases, such as the clostridial neurotoxins, proteolyse host proteins involved in neuroexocytosis, affecting neurotransmission [54]. The anthrax lethal factor, a zinc-metallopeptidase and major virulence factor of *B. anthracis*, was demonstrated to enter cells and cleave the mitogen activated protein kinase (MAPK) kinases [55]. Zmp1-like proteins, bearing similarity to an ATLF-like domain, also appear to be conserved in several bacterial species, as revealed by BlastP analyses. These also include one of the several zinc metalloproteases produced by the gut pathogen *Vibrio cholerae*, mop, which is encoded in the *Vibrio* pathogenicity island (VPI) and has been implicated in pathogenesis [56]. 

Although several metalloproteases have been predicted from the *C. difficile* genome sequence [28], none have been characterized as yet. We describe for the first time a metalloprotease, Zmp1, which demonstrates a zinc-dependent activity *in vitro*. By selectively mutating residues that are in the HEXXH motif, a conserved motif typical to zinc metalloproteases and crucial for proteolytic activity [38], we demonstrate that residues E143 and H146 are important for the catalytic activity of Zmp1. NMR and DSF analyses show that H146 is required for binding to Zn^2+^, while mutations in both residues affect activity on plasma fibronectin and fibrinogen *in vitro*. This is in accordance with the role previously attributed to these residues in other zinc metalloproteases [38,53]. In addition, our studies show that Zmp1 is active on fibrinogen Bβ-chains and fibronectin, but not on other substrates such as casein or different collagen types that were tested. Zmp1 is clearly more efficient in cleaving fibrinogen as compared to fibronectin *in vitro*, as indicated by the differences in kinetics and enzyme to substrate ratios. A low fibronectin activity may be due to the sub-optimal *in vitro* reaction conditions. As seen by the decreased activity of mutant proteins on both fibrinogen and fibronectin, the activity of Zmp1 is very specific. Furthermore, we also find that Zmp1 cleaves at the N-terminus of the Bbeta chain of fibrinogen. 

Proteolytic enzymes are frequently involved in the bacterial colonization process, contributing to nutrient acquisition, degradation of host proteins or processing of bacterial proteins involved in pathogenesis [57]. *C. difficile* strains were reported to have proteolytic activity on substrates such as collagen, casein and gelatin, although the enzymes were not identified [58]. The cysteine protease Cwp84, the best-studied *C. difficile* protease, cleaves SlpA, the precursor of high- and low-MW S-layer proteins. Cwp84 was earlier reported to cleave ECM proteins fibronectin, vitronectin and laminin [15]. We demonstrate that recombinant Zmp1 can degrade fibronectin on cultured human fibroblasts that produce a dense fibronectin network. During pathogenesis of *C. difficile*, Zmp1 may aid the bacterial colonization processes in the gut by degrading ECM components associated with the gut epithelial cells. Several pathogens including gut pathogens have been reported to secrete both proteins involved in ECM adhesion and proteases involved in ECM degradation [59]. *C. difficile* has been reported to bind to ECM associated proteins such as collagen, fibronectin and fibrinogen [60,61]. Proteases such as Zmp1 by degrading the ECM components may contribute to invasion and dissemination. Experiments examining *C. difficile* interactions with eukaryotic cells would confirm the role of Zmp1 in host cell modulation, however given the short life of cells under anaerobic conditions, such experiments are challenging. While Zmp1 demonstrates specific activity for fibrinogen and fibronectin, it is possible that it has additional ECM substrates especially in the context of gut infection. 

Thus, employing proteomics, we identify new extracellular factors that may be important for the pathogenesis of *C. difficile*. Further examination of the mechanisms involved in the release of surface-associated proteins into the extracellular milieu could clarify how the clostridial cell surface is modulated and how these proteins may interact with host cells.

## Supporting Information

Figure S1 **Proteins detected in *C. difficile* 630 and R20291 culture supernatants in individual experiments.**
(TIF)

Figure S2 **SDS_PAGE analysis of the purified Zmp1 WT, E143A and H146A.** 2.5 or 12.5µg purified recombinant protein were analyzed by SDS-PAGE followed by Coomassie-blue staining.
(TIF)

Figure S3 **Zmp1 is able to bind divalent cations.** Differential scanning fluorimetry of Zmp1 recombinant protein in the absence of divalent cations or in the presence of Zn^2+^, Cu^2+^ or Ni^2+^.
(TIF)

Figure S4 **Zinc-dependent proteolytic activity of recombinant Zmp1 on fibronectin.** 1 µM fibronectin from human plasma was incubated for 24 h at 37°C with 7.7 µM of Zmp1 in the presence of 0.5 mM ZnCl_2_, NiCl_2_, CuCl_2_ or in the absence of divalent cations. 1 µg of fibronectin was analyzed by SDS-PAGE and silver staining.
(TIF)

Figure S5 **Proteolytic activity of recombinant Zmp1 is not due to presence of contaminant *E. coli* proteases.** 1 µM fibronectin from human plasma was incubated for 24 h at 37°C with 7.7 µM Zmp1 or 5.5 µM of an unrelated protein (*Staphylococcus aureus* FhuD2) in the presence of 0.5 mM ZnCl_2_. 1 µg of fibronectin was analyzed by SDS-PAGE followed by silver staining.
(TIF)
