# Supplementary figures and images for: Identification of a Novel Zinc Metalloprotease through a Global Analysis of Clostridium difficile Extracellular Proteins
Source: PLoS One. 2013 Nov 26;8(11):e81306. doi: 10.1371/journal.pone.0081306 (PMC3841139; doi:10.1371/journal.pone.0081306)

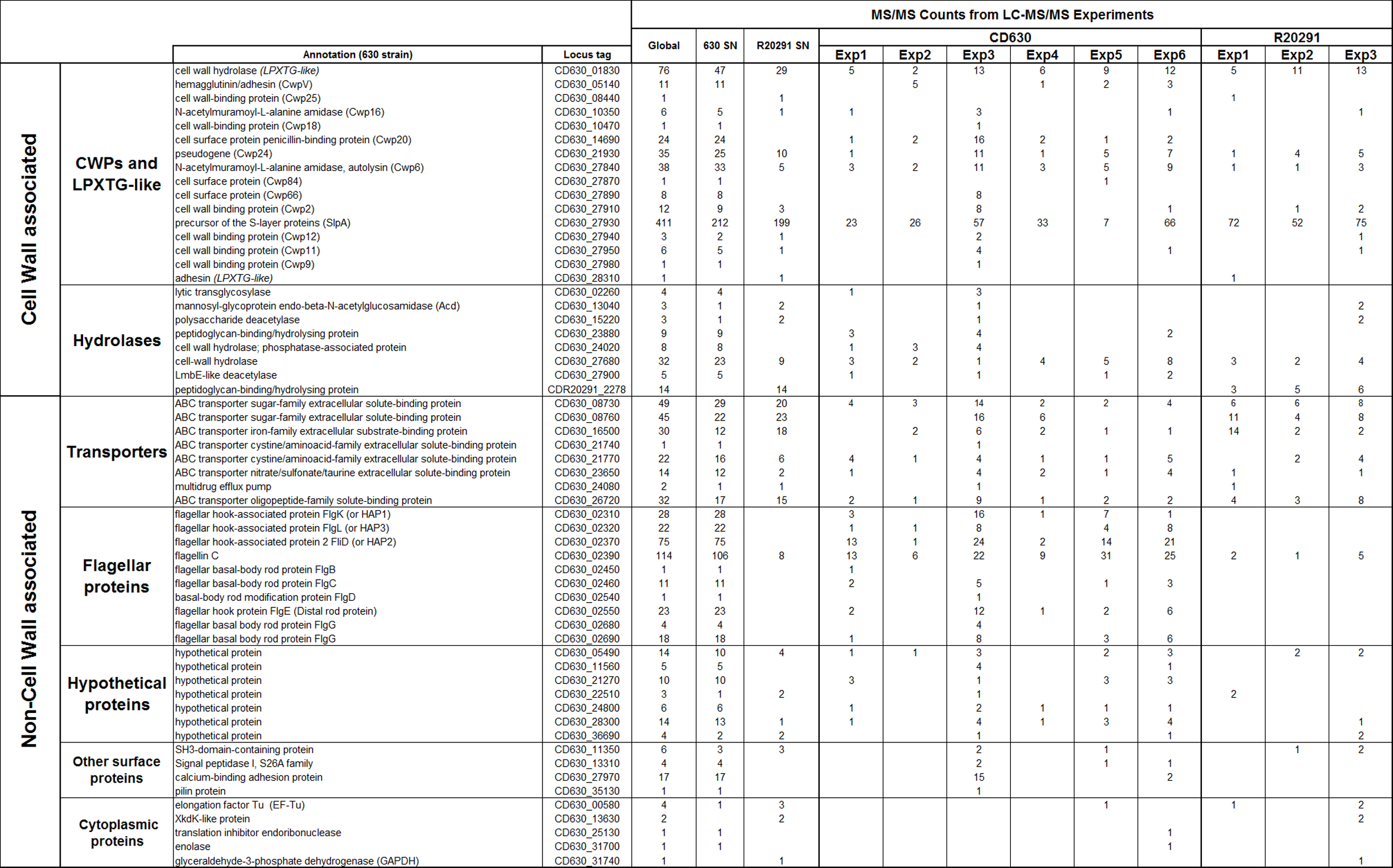

Supplement: Figure S1 — Proteins detected in C. difficile 630 and R20291 culture supernatants in individual experiments. (TIF) [file pone.0081306.s001.tif]

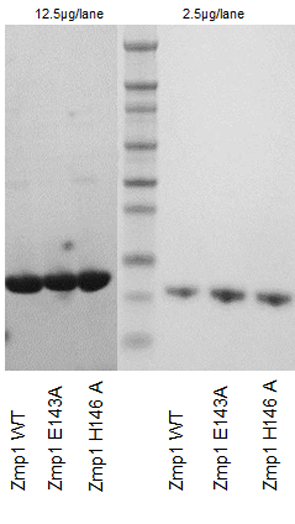

Supplement: Figure S2 — SDS_PAGE analysis of the purified Zmp1 WT, E143A and H146A. 2.5 or 12.5µg purified recombinant protein were analyzed by SDS-PAGE followed by Coomassie-blue staining. (TIF) [file pone.0081306.s002.tif]

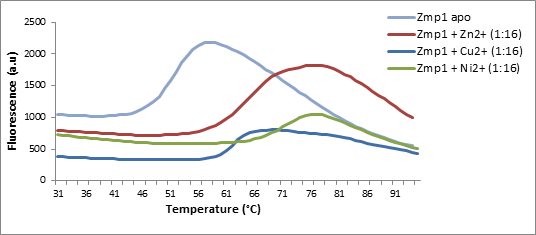

Supplement: Figure S3 — Zmp1 is able to bind divalent cations. Differential scanning fluorimetry of Zmp1 recombinant protein in the absence of divalent cations or in the presence of Zn2+, Cu2+ or Ni2+. (TIF) [file pone.0081306.s003.tif]

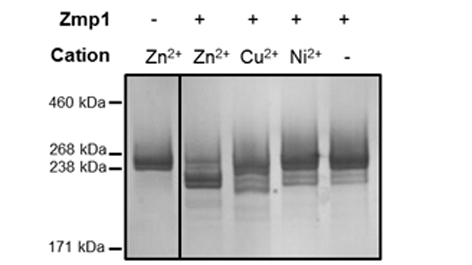

Supplement: Figure S4 — Zinc-dependent proteolytic activity of recombinant Zmp1 on fibronectin. 1 µM fibronectin from human plasma was incubated for 24 h at 37°C with 7.7 µM of Zmp1 in the presence of 0.5 mM ZnCl2, NiCl2, CuCl2 or in the absence of divalent cations. 1 µg of fibronectin was analyzed by SDS-PAGE and silver staining. (TIF) [file pone.0081306.s004.tif]

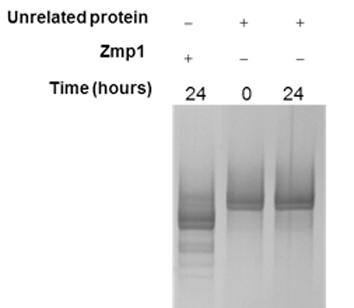

Supplement: Figure S5 — Proteolytic activity of recombinant Zmp1 is not due to presence of contaminant E. coli proteases. 1 µM fibronectin from human plasma was incubated for 24 h at 37°C with 7.7 µM Zmp1 or 5.5 µM of an unrelated protein (Staphylococcus aureus FhuD2) in the presence of 0.5 mM ZnCl2. 1 µg of fibronectin was analyzed by SDS-PAGE followed by silver staining. (TIF) [file pone.0081306.s005.tif]
